# Phytochemical and toxicological investigations of crude methanolic extracts, subsequent fractions and crude saponins of *Isodon rugosus*

**DOI:** 10.1186/0717-6287-47-57

**Published:** 2014-11-06

**Authors:** Anwar Zeb, Abdul Sadiq, Farhat Ullah, Sajjad Ahmad, Muhammad Ayaz

**Affiliations:** Department of Pharmacy, University of Malakand, Chakdara, Dir, Pakistan

**Keywords:** *Isodon rugosus*, Cytotoxicity, Phytotoxicity, Brine shrimps

## Abstract

**Background:**

*Isodon rugosus* is used traditionally in the management of hypertension, rheumatism, tooth-ache and pyrexia. Present study was arranged to investigate *I. rugosus* for phytoconstituents, phytotoxic and cytotoxic activities to explore its toxicological, pharmacological potentials and to rationalize its ethnomedicinal uses. Briefly, qualitative phytochemical analysis of the plant extracts were carried out for the existence of alkaloids, flavonoids, saponins, oils, glycosides, anthraquinones, terpenoids, sterols and tannins. Plant crude methanolic extract (Ir.Cr), its subsequent fractions; *n*-hexane (Ir.Hex), chloroform (Ir.Chf), ethyl acetate (Ir.EtAc), aqueous (Ir.Aq) and saponins (Ir.Sp) in different concentrations were tested for phytotoxic and cytotoxic activities using radish seeds and brine shrimps (*Artemia salina*) respectively. The phytotoxic activity was determined by percent root length inhibition (RLI) and percent seeds germination inhibition (SGI) while the cytotoxicity was obtained with percent lethality of the brine shrimps.

**Results:**

Ir.Cr was tested positive for the presence of alkaloids, glycosides, flavonoids, oils, terpenoids, saponins, tannins and anthraquinones. Among different fractions Ir.Sp, Ir.Chf, Ir.EtAc, and Ir.Cr were most effective causing 93.55, 89.32, 81.32 and 58.68% inhibition of seeds in phytotoxicity assay, with IC_50_ values of 0.1, 0.1, 0.1 and 52 μg/ml respectively. Similarly, among all the tested samples, Ir.Sp exhibited the highest phytotoxic effect causing 91.33% root length inhibition with IC_50_ of 0.1 μg/ml. Ir.Sp and Ir.Chf were most effective against brine shrimps showing 92.23 and 76.67% lethality with LC_50_ values of 10 and 12 μg/ml respectively.

**Conclusions:**

It may be inferred from the current investigations that *I. rugosus* contains different secondary metabolites and is a potential source for the isolation of natural anticancer and herbicidal drug molecules. Different fractions exhibited phytotoxic and cytotoxic activities, thus providing pharmacological basis for ethnomedicinal uses of this plant.

## Background

The human environment and life are at constant risk of various complications [1]. Every individual on earth is in search of clean and healthy time. For the management of these major obstacles, a constant utilization of synthetic resources is the common inspiration [2]. Synthetic resources, being the most helpful tools to get rid of many complications are also a great cause of severe side effects [3–5]. So it is obvious that exploring the natural sources will be a great step toward the solution.

According to a survey, medicinal plants cover 34% of the total plant species found in Pakistan [6]. In Pakistan at least six thousand flowering plants have been reported currently, among which 400–600 are of great medicinal importance [7]. Herbs have been employed in variety of medicinal and non-medicinal purposes, like flavoring, nutrition, spices, beverages, dyeing, repellents, cosmetics, perfumery, fuels and in many other industrial uses. Worldwide about 85% of primary health care medications are depend on natural sources [8]. Till the nineteenth century, man has discovered the great contribution of herbs in the management of almost every pharmacological disorder [9]. According to an estimation, up to 70,000 plant species are used ethno-medicinally worldwide [10]. Sidewise, there are several factors which influence the plant production, such as pest and weeds [11, 12].

Plants in the same family exhibit almost a similar pharmacological profile due to the same genome [13]. The plants in family labiateae or lamiaceae are explored for various pharmacological purposes, like analgesic, anthelmintic and antibacterial [14]. *I. rugosus* is used ethno-medicinally in the diseases of skin, ear, nose, throat and intestine [15]. Basically the Phytochemicals are broadly classified into two main categories, namely, primary constituents and secondary metabolites. Primary constituents includes proteins, amino acids,, common sugars and chlorophyll, whereas, secondary constituents include glycosides, alkaloids, phenolic compounds, flavonoids, saponins, essential oils, tannins and terpenoids [16]. Majority of phytochemicals contain important therapeutic activities and the plants thus find their medicinal importance due to presence of the respective phytochmical constituents. Furthermore, each pharmacological action demonstrated by a plant sample is attributed to the presence of secondary metabolites in it. In this regard possibly interesting class of molecules are the saponins, secondary plant metabolites composed of steroids or triterpenoid aglycone linked by glycosidic bonds with divergent biological activities [17]. Saponins having the ability to treat different disease conditions and have been used as antimicrobial, antidiabetic, cytotoxic, phytotoxic, antitumor, antioxidant, antispasmodic and as anthelmintic [18–21]. The crude saponins have been studied for their anticancer [22], anthelmintic [23] and insecticidal potentials [24].

Keeping in view the published importance of saponins and exploring the medicinal importance of *I. rugosus*, we have achieved its phytochemical studies along with phytotoxic and cytotoxic potentials.

## Results

### Phytochemical analysis

Ir.Cr was found to contain glycosides, alkaloids, tannins, terpenoids, flavonoids, saponins, anthraquinones and oils, while tested negative for the presence of sterols (Table 1).

**Table 1:** **Phytochemical constituents in crude extract of** ***I. rugosus***

S. no	Phytochemicals	Observations	Results
**1**	Alkaloids	Turbidity/precipitation	**+**
**2**	Flavonoids	Formation of yellow color which changed to colorless on acid addition	**+**
**3**	Saponins	Formation of frothing bubbles	**+**
**4**	Tannins	Formation of bluish-black color	**+**
**5**	Sterols	green to pink color was absent	**-**
**6**	Glycosides	Formation of red color precipitate	**+**
**7**	Anthraquinones	Formation of red, violet or pink color in aqueous layer	**+**
**8**	Terpenoids	Appearance of reddish brown color	**+**
**9**	Oils	Formation of Greasy spot	**+**

Positive symbol (+) shows the presence while negative symbol (-) shows the absence of phytochemicals.

### Phytotoxic assay

The radish seed germination assay is of great importance for the phytotoxic activity of plant extract. The activity involves the measurement of the average root length and number of seeds inhibition in which the water serves as negative control. All the tested samples for phytotoxic potential of *I. rugosus* are summarized in Table 2. In this study the Ir.Sp revealed significant phytotoxicity i.e., root length inhibition (RLI) (IC_50_ = 0.1 μg/ml) and seeds germination inhibition (SGI) (IC_50_ = 0.1 μg/ml). Among the fractions Ir.Chf exhibited highest phytotoxic activity i.e., 88.40 ± 1.36, 71.37 ± 0.74 and 66.51 ± 0.39% (IC_50_ = 0.1 μg/ml) RLI and 89.32 ± 3.52, 73.59 ± 0.58 and 68.37 ± 0.62% (IC_50_ = 0.1 μg/ml) SGI at the concentration of 1000, 100 and 10 μg/ml respectively. Ir.EtAc demonstrated 80.40 ± 0.* + -68, 74.55 ± 0.51 and 61.55 ± 0.67% (IC_50_ = 0.1 μg/ml) RLI and 81.32 ± 5.8, 71.55 ± 0.65 and 59.44 ± 0.58% (IC_50_ = 0.1 μg/ml) SGI at 1000, 100 and 10 μg/ml respectively. The Ir.Hex showed 80.40 ± 0.68, 77.33 ± 0.64 and 47.26 ± 0.64% (IC_50_ = 29 μg/ml) RLI and 56.00 ± 4.6, 47.26 ± 0.61 and 41.55 ± 0.61% (IC_50_ = 410 μg/ml) SGI at 1000, 100 and 10 μg/ml respectively. The Ir.Cr and Ir.Aq revealed moderate phytotoxicity. The order of phytotoxicity for the given fractions were Ir.Chf > Ir.EtAc > Ir.Cr > Ir.Hex > Ir.Aq. From scientific verification it may be inferred that the other plants growing nearby *I. rugosus* may affected their growth. Majorly the phenolics are responsible for the phytotoxic action of plant [25].

**Table 2:** **Phytotoxic effect of the crude extract of** ***I. rugosus*** **and its fractions against radish seeds**

Samples	Conc. (μg/ml)	Root length inhibition% (mean ± SEM)	IC _50_ (μg/ml)	Seeds inhibition% (mean ± SEM)	IC _50_(μg/ml)
**Ir.Cr**	1000	72.00 ± 0.20^***^	25	58.68 ± 3.52^***^	52
	100	63.15 ± 0.91^***^		54.49 ± 0.47^***^	
	10	45.15 ± 0.61^***^		39.51 ± 0.58^***^	
**Ir.Hex**	1000	80.40 ± 0.68^***^	29	56.00 ± 4.60^***^	410
	100	77.33 ± 0.64^***^		47.26 ± 0.61^***^	
	10	47.26 ± 0.64^***^		41.55 ± 0.61^***^	
**Ir.Chf**	1000	88.40 ± 1.36^*^	0.1	89.32 ± 3.52 ^ns^	0.1
	100	71.37 ± 0.74^***^		73.59 ± 0.58 ^ns^	
	10	66.51 ± 0.39^*^		68.37 ± 0.62 ^ns^	
**Ir.EtAc**	1000	80.40 ± 0.68^***^	0.1	81.32 ± 5.80 ^ns^	0.1
	100	74.55 ± 0.51^***^		71.55 ± 0.65^*^	
				59.44 ± 0.58^**^	
	10	61.55 ± 0.67^***^			
**Ir.Aq**	1000	40.40 ± 0.44^***^	1830	49.32 ± 3.52^***^	1045
	100	33.67 ± 0.55^***^		41.55 ± 0.64^***^	
	10	30.55 ± 0.64^***^		33.62 ± 0.61^***^	
**Ir.Sp**	1000	91.33 ± 0.62	0.1	93.55 ± 0.64	0.1
	100	84.52 ± 0.48		81.44 ± 0.67	
	10	69.51 ± 0.79		66.59 ± 0.52	
**Negative control**		–		–	

Results expressed as average seeds inhibition and Inhibition of root length (mean ± SEM n = 3) and IC_50_. Values significantly different as compared to Standard drug; *P <0.05, **P <0.01, ***P <0.001.

### Cytotoxic assay

The cytotoxic effect exhibited by Ir.Cr, Ir.Sp and resultant fractions of *I. rugosus* is presented in Table 3. Lethality was highest at the concentration of 1000 μg/ml while low at the minimum concentration of each fraction. The Ir.Sp showed highest cytotoxic activity against brine shrimps i.e., 92.23 ± 1.1, 66.67 ± 1.9 and 52.23 ± 1.1% brine shrimps lethality at the concentration of 1000, 100 and 10 μg/ml respectively with the LC_50_ of 10 μg/ml. Ir.Chf showed (76.67 ± 3.3, 62.23 ± 2.9 and 48.90 ± 1.1)% cytotoxic effect at the concentration of 1000, 100, 10 μg/ml respectively with the LC_50_ of 12 μg/ml. The Ir.EtAc also showed almost a similar cytotoxicity (74.43 ± 2.2, 57.77 ± 2.2 and 43.33 ± 1.9)% at the concentration of 1000, 100, 10 μg/ml respectively with the LC_50_ of 315 μg/ml. The Ir.Aq also showed slightly lower cytotoxicity (65.57 ± 1.1, 53.33 ± 1.9 and 42.23 ± 1.1)% at the concentration of 1000, 100, 10 μg/ml respectively with the LC_50_ of 60 μg/ml. The cytotoxic profile of Ir.Hex and Ir.Cr were lower compared to other fractions. The LC_50_ calculated were 10, 12, 60, 120, 220 and 315 μg/ml for Ir.Sp, Ir.Chf, Ir.Aq, Ir.Hex, Ir.Cr and Ir.EtAc respectively. The order of cytotoxic activity were Ir.Sp > Ir.Chf > Ir.EtAc > Ir.Aq > Ir.Hex > Ir.Cr.

**Table 3:** **Concentration dependent cytotoxic effect of the Ir.Cr extract of** ***I. rugosus*** **and its fractions against Artemia salina**

Extracts	Total treated	Dose (μg/ml)	% Lethality (mean ± SEM)	LC _50_(μg/ml)
**Ir.Cr**	30	1000	71.10 ± 1.1^***^	220
		100	46.67 ± 3.3^***^	
		10	37.77 ± 1.1^***^	
**Ir.Hex**	30	1000	62.23 ± 2.9^***^	120
		100	48.90 ± 1.1^***^	
		10	40.00 ± 1.9^***^	
**Ir.Chf**	30	1000	76.67 ± 3.3^***^	12
		100	62.23 ± 2.9 ^ns^	
		10	48.90 ± 1.1 ^ns^	
**Ir.EtAc**	30	1000	74.43 ± 2.2^***^	315
		100	57.77 ± 2.2^*^	
		10	43.33 ± 1.9^*^	
**Ir.Aq**	30	1000	65.57 ± 1.1^***^	60
		100	53.33 ± 1.9^***^	
		10	42.23 ± 1.1^**^	
**Ir.Sp**	30	1000	92.23 ± 1.1	10
		100	66.67 ± 1.9	
		10	52.23 ± 1.1	

Standard drug; Etoposide, LC_50_ = 9.8 μg/ml. Values represents % lethality and are expressed as mean ± SEM; *P <0.05, **P <0.01, ***P <0.001.

## Discussion

Cytotoxic activity is one of the prominent assay to develop novel anticancer drug candidates in different plants samples [26]. In the present study, *I. rugosus* is investigated for the determination of its anticancer potential against Artemia salina. The order of brine shrimps lethality is crude Ir.Sp > Ir.Chf > Ir.EtAc > Ir.Aq > Ir.Hex > Ir.Cr. The cytotoxic effect of Ir.Sp and Ir.Chf were the most prominent. The high lethality of these plant samples showed that substances responsible for the cytotoxic effect are present in high amount in crude Ir.Sp and Ir.Chf fraction. As reported earlier that a positive co-relation exists between the brine shrimp lethality assay and human nasopharyngeal carcinoma (KB cell line) [27], so this method was employed for the determination of possible antineoplastic activity.

The phytotoxic activity of *I. rugosus* was carried out by employing radish seeds. The radish root length inhibition and seeds germination inhibition is actually due to phytotoxic effect of *I. rugosus*. The radish seeds growth inhibitin was highest for Ir.Chf fraction while lowest for the Ir.Aq as shown in the Table 2. Comparatively, the Ir.EtAc fraction showed low phytotoxicity as compared to Ir.Chf fraction. The other fractions also showed moderate phytotoxic effect which was comparatively low from that of Ir.Chf and Ir.EtAc. It is deduced from the results that the Ir.Chf and Ir.EtAc fractions of *I. rugosus* contain high amount of that constituents which are responsible for phytotoxic effect. Hence, it is deduced that constituents of the plant with herbicidal action are highly concentrated in Ir.Chf fraction.

Weeds play an important role in low production of crops. Huge economic loss in agriculture is reported each year due to harmful effects of weeds. Different techniques and chemicals are used to eliminate the harmful effects of these weed [28]. But there application is limited due to their other health hazard such as food toxicity and economical factors [29]. Hence alternative factors should be considered for removing these unwanted effects, which may be economical and having minimum harmful effects. The natural herbicides are being investigated in plants to get rid of the delayed hazardous effects of synthetic herbicides [30]. The Natural herbicides are preferred over chemical agents due to safety profile and low cost.

The Ir.Sp obtained from *I. rugosus* are assayed for the cytotoxic activity because saponins are reported to have anticancer action [31]. Saponins are the active constituent verified by many researchers for its anticancer potentials applying antitumor activity using potato model [32]. The brine shrimps lethality assay has also been conducted on Ir.Sp and have been resulted in promising lethality and hence proved to have cytotoxic activity [33]. The results of this activity reveal that constituents responsible for cytotoxic and phytotoxic effect of *I. rugosus* may be a significant source of natural herbicides for weeds to enhance the crops production rate in the field of agriculture.

## Conclusion

This study reveals that *I. rugosus* contain secondary metabolites like; glycosides, alkaloids, saponins, tannins, flavonoids, terpenoids and anthraquinones. The plant crude extract, its subsequent fractions and crude saponins exhibited concentration dependent phytotoxic and cytotoxic actions which warrant isolation and characterization of novel and cost-effective compounds responsible for such valuable activities.

## Methods

### Collection and extraction

The plant was collected from Dir (KPK), Pakistan in the month of June. The plant name was confirmed at the Department of Botany, Shaheed Benazir Bhuto University Dir (KPK), Pakistan. The plant sample was kept in the herbarium of the same university with voucher number (1016AZ). The fresh aerial parts of plant were cleaned with distilled water and kept at room temperature for drying. After shade drying, the plant was grinded to coarse powder with the help of cutter mill. The powdered material (8 kg) was kept for maceration in 30 liters of 80% methanol with daily base occasional shaking for 22 days. It was then filtered and concentrated under reduced pressure using rotary evaporator at 40°C. Finally, keeping the concentrated sample in water bath a semisolid crude extract was obtained with weight of 300 g [34].

### Fractionation

The Ir.Cr extract was suspended in 500 ml of distilled water and transferred into a separating funnel followed by the addition of an equal amount of *n*-hexane. Shake it vigorously and then kept for a while to separate the two layers. Both the layers were separated. Similarly, the fractionation was carried out by successive solvent-solvent extraction with increasing polarity of the solvent. Each separated fraction was concentrated individually under reduced pressure using rotary evaporator. The fractions obtained were Ir.Hex 22 g, Ir.Chf 35 g, Ir.EtAc 80 g and Ir.Aq fraction of 120 g.

### Extraction of crude Saponins

To the pulverized plant sample (20 g) was added 100 ml of 20% ethanol and kept in water bath for 4 h at 55°C. The sample was filtered and extracted again with 200 ml of 20% ethanol. Then by keeping it in water bath for some time, the volume dropped down to 40 ml. The sample was then transferred into a separating funnel and added 20 ml diethyl ether to it with vigorous shaking. Both the diethyl ether and aqueous layers were separated. The aqueous layer was mixed with 60 ml of *n*-butanol and separated by using a separating funnel. The *n*-butanol extract was washed with 10 ml of 5% brine solution. The final volume was concentrated in a water bath and then transferred into a beaker. The sample was kept in an oven to get dried Ir.Sp weighing 1.1 g [35].

### Phytochemical investigation

Qualitative phytochemical analysis of the plant extracts was carried out for the existence of glycosides, flavonoids, saponins, alkaloids, anthraquinones, terpenoids, sterols and tannins using the reported procedures [36]. For the detection of glycosides the plant extracts were hydrolyzed with HCl followed by neutralization with NAOH. Few drops of Fehling’s solution were added to the preparation and determined by the presence of red precipitates. The alkaloids were determined by using Dragendorff’s reagent. For the detection of terpenoids and sterols the samples were treated with petroleum ether followed by extraction with chloroform. The appearance of reddish brown color for terpenoids and green to pink color for sterols was noted after treatment of chloroform layer with acetic anhydride and concentrated HCl in series. Anthraquinones were determined by the process of dissolving the extract in 1% of HCl, then benzene and finally mixing with NH_4_OH. The presence of anthraquinones was observed by appearance of pink, red or violet color.

The Presence of saponins was detected by the formation of froth bubbles in a beaker upon vigorous shaking using diluted samples.

### Phytotoxicity (radish seeds) assay

The stock solution of each extract was prepared according the reported procedure [37]. A specific quantity, i.e. 50 mg of each extract was dissolved in 5 ml of methanol and 5 ml of *n*-hexane respectively in a beaker to obtain concentration of 10,000 μg/ml. Proper size filter papers (Whatman #1) were kept in the petri plates. The solution containing plant extract was added to the petri dishes. By keeping in laminar flow for a while the solvent get evaporated from the petri plates. Each petri dish was diluted with 5 ml of distilled water. Methanol, *n*-hexane and distilled water were used as control group. The seeds were sterilized with 0.1% mercuric chloride solution. Twenty five radish seeds were placed in each petri plate. All the petri plates containing the seeds were kept in incubator for five days at 25°C. The root length and number of seeds germination inhibition were measured and counted. All the tests were performed in triplicate.

### Cytotoxicity (Brine shrimps lethality) assay

The cytotoxic effect against Artemia salina (brine shrimp eggs) of the Ir.Cr and various fractions (Ir.Hex, Ir.Chf, Ir.EtAc, Ir.Aq) and Ir.Sp were carried following the reported procedure [38].

Brine solution was prepared by dissolving 38 g of sodium chloride in double distilled water. The brine solution prepared was simulated sea water. A tray having dimensions of 22 × 32 cm was used in which a partition was created by a perforated plate. One side of the tray was covered by aluminum foil in which 50 mg Artemia salina eggs were sprinkled and the other side was kept as such to be the place for newly hatched brine shrimps larvae. The tray was kept in incubator at 37°C for hatching purpose. After 24 h when the larvae got hatched, it was attracted from dark side using torch and collected using a Pasteur pipette. 30 larvae were put in different vials. Three dilutions of the solution were made by dissolving 20 mg of the test sample in 2 ml of organic solvents and then by transferring 5, 50 and 500 μl of the stock solution into three different vials making 10, 100 and 1000 μg/ml concentrations respectively. To each vial sea water was added to adjust the volume 5 ml. Etoposide was used as positive control while for negative control sea water was used. The brine shrimps were kept in the vials for 24 h under light at 25°C. After 24 h, the number of brine shrimps alive and dead was counted in each vial using magnifying glass. The data was recorded in triplicate with the determination of LC_50_.

### Statistical analysis

Each experiment was performed in replicates and values were expressed as mean ± SEM. Two way ANOVA followed by multiple comparison Bonferroni’s test were used to compare control group with the tested groups. The P values less than 0.05 were considered as statistically significant. IC_50_ values were calculated by a linear regression analysis among the percent inhibition against the extract concentrations via Microsoft Office Excel 2007 program.
